# AgNa_2_Mo_3_O_9_AsO_4_

**DOI:** 10.1107/S1600536811041961

**Published:** 2011-10-22

**Authors:** Hamadi Hamza, Mohamed Faouzi Zid, Ahmed Driss

**Affiliations:** a Laboratoire de Matériaux et Cristallochimie, Faculté des Sciences, Université de Tunis–ElManar, 2092 El-Manar, Tunis, Tunisia

## Abstract

The title compound, silver disodium trimolybdenum(VI) nonaoxide arsenate, AgNa_2_Mo_3_O_9_AsO_4_, was prepared by a solid-state reaction at 808 K. The structure consists of an infinite (Mo_3_AsO_13_)_*n*_ ribbon, parallel to the *c* axis, composed of AsO_4_ tetra­hedra and MoO_6_ octa­hedra sharing edges and corners. The Na and Ag ions partially occupy several independent close positions, with various occupancies, in the inter-ribbon space delimited by the one-dimensional framework. The composition was refined to Ag_1.06(1)_Na_1.94(1)_Mo_3_O_9_AsO_4_.

## Related literature

For framework structures containing *M*O_6_ and *X*O_4_ (*M* = transition metal, *X* = P, As) building blocks, see: Benhamada *et al.* (1992 ▶); Harrison *et al.* (1994 ▶); Guyomard *et al.* (1999 ▶); Ben Smail *et al.* (1999 ▶); Ben Amor & Zid (2006 ▶). For a similar one-dimensional structure, see: Hamza *et al.* (2010 ▶). For details of the synthesis, see: Hajji *et al.* (2004 ▶, 2005 ▶); Ben Hlila *et al.* (2009 ▶); Zid & Jouini (1996 ▶); Zid *et al.* (1998 ▶). For the bond-valence model, see: Brown & Altermatt (1985 ▶). For physical properties of related compounds, see: Daidouh *et al.* (1997 ▶); Ouerfelli *et al.* (2004 ▶, 2007 ▶); Piffard *et al.* (1985 ▶); Oyetola *et al.* (1988 ▶); Goubitz *et al.* (2001 ▶); Ledain *et al.* (1997 ▶); Harrison *et al.* (1998 ▶); Hajji & Zid (2006 ▶); Ruiz *et al.* (2002 ▶).

## Experimental

### 

#### Crystal data

AgNa_2_Mo_3_O_9_AsO_4_
*M* *_r_* = 729.68
Triclinic, 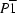

*a* = 8.1767 (8) Å
*b* = 9.7687 (9) Å
*c* = 8.0451 (8) Å
α = 99.49 (2)°
β = 106.07 (2)°
γ = 113.29 (2)°
*V* = 539.14 (19) Å^3^
*Z* = 2
Mo *K*α radiation
μ = 8.50 mm^−1^
*T* = 298 K
0.36 × 0.24 × 0.16 mm

#### Data collection

Enraf–Nonius CAD-4 diffractometer
Absorption correction: ψ scan (North *et al.*, 1968 ▶) *T*_min_ = 0.098, *T*_max_ = 0.247
2853 measured reflections
2355 independent reflections
2047 reflections with *I* > 2σ(*I*)
*R*_int_ = 0.023
2 standard reflections every 120 min  intensity decay: 1.1%

#### Refinement

*R*[*F* ^2^ > 2σ(*F* ^2^)] = 0.024
*wR*(*F* ^2^) = 0.063
*S* = 1.09
2355 reflections
188 parameters
3 restraints
Δρ_max_ = 0.95 e Å^−3^
Δρ_min_ = −0.76 e Å^−3^

### 

Data collection: *CAD-4 EXPRESS* (Enraf–Nonius, 1994 ▶); cell refinement: *CAD-4 EXPRESS*; data reduction: *XCAD4* (Harms & Wocadlo, 1995 ▶); program(s) used to solve structure: *SHELXS97* (Sheldrick, 2008 ▶); program(s) used to refine structure: *SHELXL97* (Sheldrick, 2008 ▶); molecular graphics: *DIAMOND* (Brandenburg, 1998 ▶); software used to prepare material for publication: *WinGX* (Farrugia, 1999 ▶).

## Supplementary Material

Crystal structure: contains datablock(s) I, global. DOI: 10.1107/S1600536811041961/br2178sup1.cif

Structure factors: contains datablock(s) I. DOI: 10.1107/S1600536811041961/br2178Isup2.hkl

Additional supplementary materials:  crystallographic information; 3D view; checkCIF report

## Figures and Tables

**Fig. 1.**
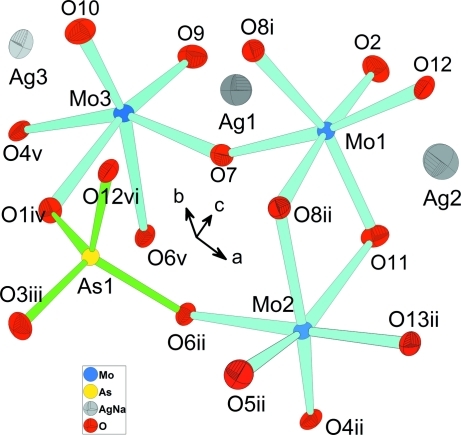
: Unité asymétrique dans Na2AgMo3O9AsO4. Les élipsoides sont définis avec 50% de probabilité. Code de Symétrie: (i)1 - x, 1 - y, 1 - z; (ii)x, -1 + y, z; (iii)x, -1 + y, -1 + z; (iv)x, y, -1 + z; (v)1 - x, 1 - y, -z; (vi)1 - x, -y, 1 - z.

**Fig. 2.**
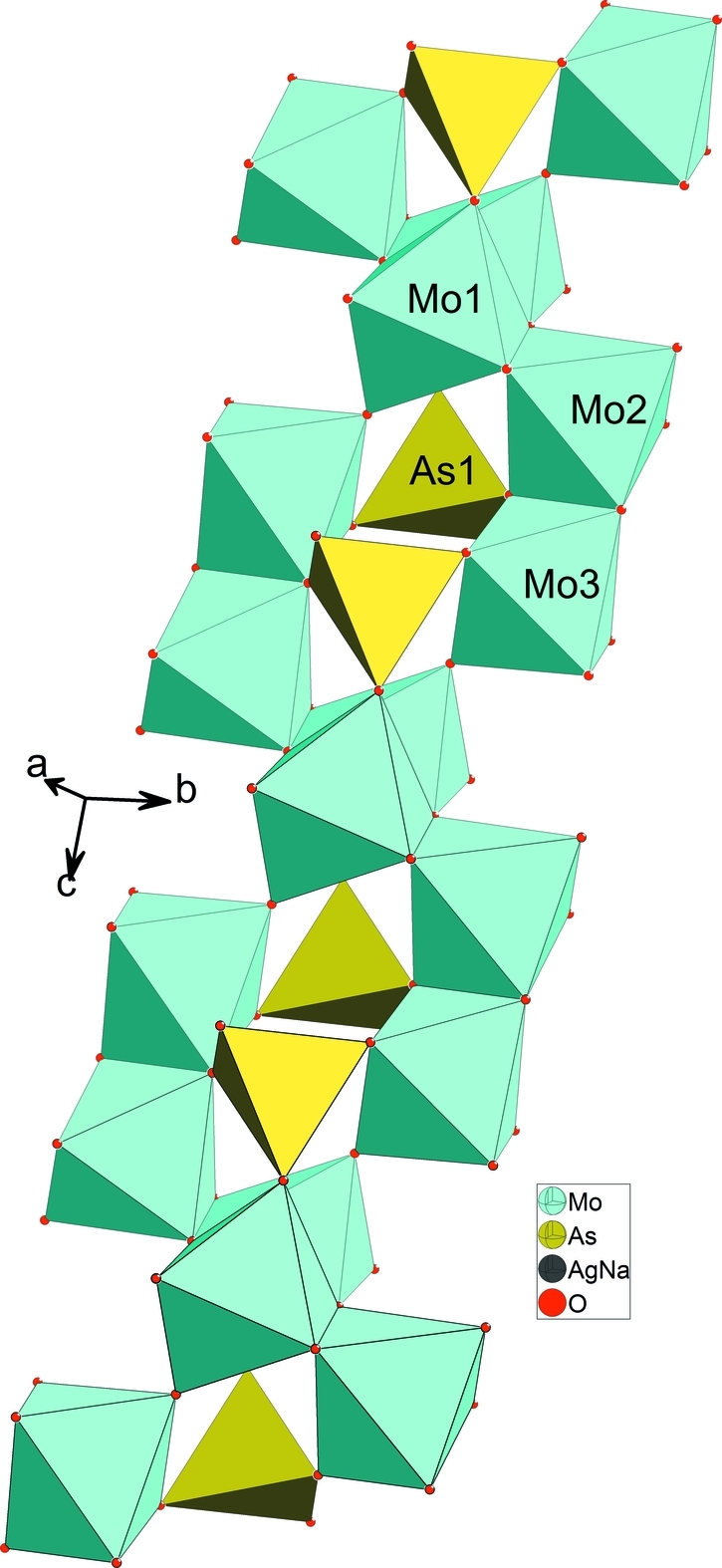
: Projection d'un ruban de Na2AgMo3O9AsO4, mettant en évidence les jonctions des polyèdres.

**Fig. 3.**
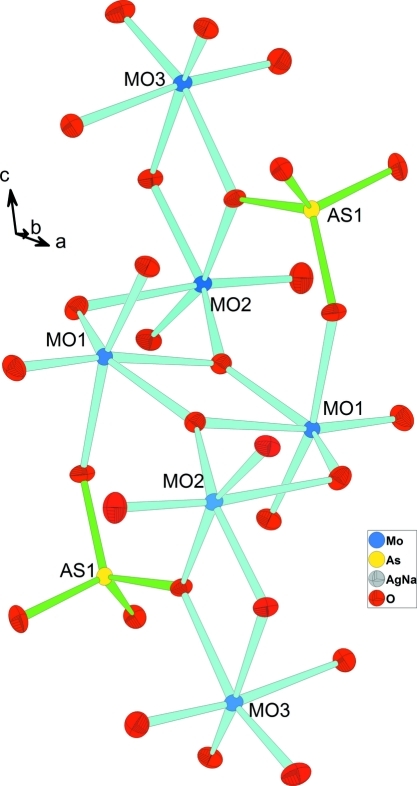
: Vue en perspective d'un ruban mettant en évidence les deux types de ponts triples dans la structure.

**Fig. 4.**
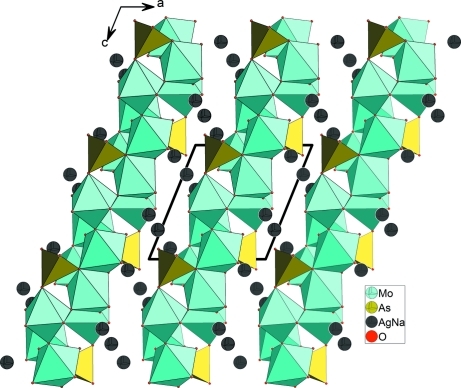
: Projection de la structure de Na2AgMo3O9AsO4, selon b, mettant en évidence les espaces inter-rubans où résident les cations.

**Fig. 5.**
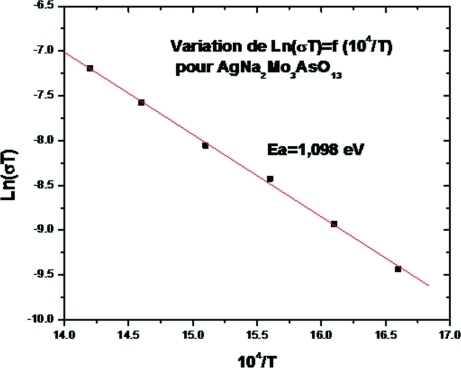
: Conductivité ionique en fonction de la température pour Na2AgMo3O9AsO4.

**Fig. 6.**
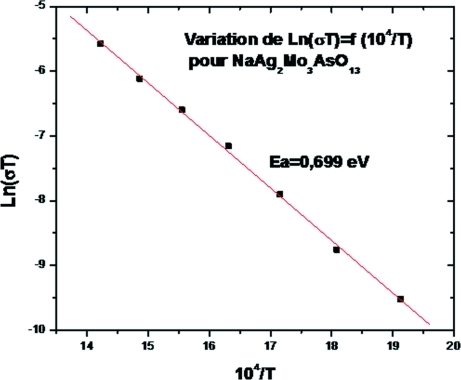
: Conductivité ionique en fonction de la température pour Ag2NaMo3O9AsO4.

## supplementary crystallographic information

### Comment

La recherche de nouveaux matériaux à charpentes mixtes formées d'octaèdres MO_6_ (*M* = métal de transition) et de tétraèdres XO_4_ (*X* = P, As) suscite un grand intérêt ces dernières années (Guyomard *et al.*, 1999; Benhamada *et al.*, 1992; Harrison *et al.*, 1994; Ben Smail *et al.* 1999; Ben Amor & Zid, 2006). En effet, la jonction entre ces polyèdres conduit à des composés à charpentes ouvertes présentant de nombreuses propriétés physico-chimiques intéressantes qui sont en relation directe avec leurs structures cristallines notamment: conduction ionique (Piffard *et al.*, 1985), échange d'ions (Oyetola *et al.*, 1988) et parfois comme produits d'intercalation en catalyse hétérogène (Goubitz *et al.*, 2001; Ledain *et al.*, 1997). C'est dans ce cadre que nous avons exploré les systèmes A—Mo—As—O (A= cation monovalent) dans lesquels nous avons précédemment caractérisé les phases suivantes: K_2_MoAs_2_O_9_ (Zid & Jouini, 1996), Rb_2_MoAs_2_O_9_ (Zid *et al.*, 1998), β-LiMoAsO_6_ (Hajji *et al.*, 2004), LiMo_2_AsO_9_ (Hajji *et al.*, 2005) et β-NaMoAsO_6_ (Ben Hlila *et al.*, 2009). A fin d'augmenter la mobilité des cations en passant à une occupation partielle des sites dans la structure nous avons choisi d'introduire avec le cation alcalin un métal monovalent de transition Ag^+^. Dans ce travail nous nous sommes intéressés en premier à la synthèse et l'étude structurale sur monocristal du matériau puis à l'étude de l'influence de l'introduction d'un métal de transition monovalent sur ces propriétés physiques notamment de conduction ionique. Le composé Na_2_AgMo_3_O_9_AsO_4_ obtenu est de formulation et de symétrie similaires à celles de NaAg_2_Mo_3_O_9_AsO_4_ (Hamza *et al.*, 2010).

L'unité asymétrique du composé Na_2_AgMo_3_O_9_AsO_4_ peut être decrite au moyen de l'unité classique MoAsO_8_ reliée par mize en commun de sommets à un un groupement Mo_2_O_10_ formé à partir d'une paire d'octaèdres MoO_6_ partageant une arête (Fig. 1).

La structure du composé Na_2_AgMo_3_O_9_AsO_4_ est construite à partir de rubans infinies (Mo_3_AsO_13_)_n_ disposés selon la direction [001] (Fig. 2). Notons qu'au sein de la charpente anionique (Mo_3_O_9_AsO_4_)^2-^, les octaèdres MoO_6_ se lient par partage d'arêtes formant des groupements (Mo_3_O_14_) qui par mize en commun de sommets developpent des unités cycliques à six octaèdres. De plus, les atomes d'oxygène formant l'arête commune de certaines paires d'octaèdres appartiennent aussi soit aux tétraèdres AsO_4_ soit aux octaèdres Mo2O_6_ (Fig. 3). Il en résulte ainsi une charpente uni-dimensionnelle, similaire à celle rencontée dans le composé Ag_2_NaMo_3_O_9_AsO_4_ (Hamza *et al.*, 2010), possédant des espaces inter-rubans où résident les cations monovalents (Fig. 4). L'examen des facteurs géométriques dans la structure montre qu'ils sont conformes à ceux rencontrés dans la littérature (Hajji & Zid, 2006; Ben Hlila *et al.*, 2009). De plus, l'utilization de la méthode BVS, pour le calcul des différentes valences des liaisons, utilisant la formule empirique de Brown (Brown & Altermatt, 1985) vérifie bien les valeurs de charges des ions attendues dans la phase étudiée: Mo1 (5,942), Mo2 (5,974), Mo3 (5,909) et As1 (4,844). La répartition des cations sur plusieurs positions et leur occupation partielle des sites, pourraient conduire à une forte mobilité, par conséquent à un nouveau conducteur ionique (Daidouh *et al.*, 1997; Ruiz *et al.*, 2002; Ouerfelli *et al.*, 2007). En effet, des mesures électriques moyennant un pont d'impédance complexe de type HP4192A ont été réalisées sur une phase pure de Na_2_AgMo_3_O_9_AsO_4_ compactée sous forme de pastille. L'ensemble pastille-électrodes constitue une cellule électrochimique équivalente à deux circuits électriques de type RC montés en série et caractérisés par leur impédance. Par ailleurs la variation de la conductivité ionique en fonction de la température suit la loi d'Arrhenius (Fig. 5): Ln(σT) = f(10^4^/T). Le calcul de la pente de la droite permet d'accéder à la valeur de l'énergie d'activation Ea = 1,06 eV, celle de la conductivité ionique à 430°C s'avère de l'ordre de 1,387 10^-6^ S.cm^-1^. La comparaison de ces valeurs à celles trouvées expérimentalement pour le composé Ag_2_NaMo_3_O_9_AsO_4_ (Fig 6): Ea = 0,699 eV e t de la conductivité ionique à 430°C égale à 5,387 10^-6^ S.cm^-1^, montre une amélioration de la valeur de la conductivié et un gain dans la largeur de la bande interdite Ea, au cours du remplacement, dans la structure, de l'ion sodium par Ag^+^, de taille similaire mais ion plus polarisable. Ces résultats permettent de classer, en se basant sur la littérature, les deux matériaux synthétisés Na_2_AgMo_3_O_9_AsO_4_ et Ag_2_NaMo_3_O_9_AsO_4_ (Hamza *et al.*, 2010) dans la famille des conducteurs ioniques moyens (Daidouh *et al.*, 1997; Harrison *et al.* 1998; Hajji & Zid, 2006; Ouerfelli *et al.*, 2004).

### Experimental

Les cristaux relatifs à AgNa_2_Mo_3_O_9_AsO_4_ ont été obtenus à partir d'un mélange formé des réactifs: (NH_4_)_2_Mo_4_O_13_ (Fluka, 69858), NH_4_H_2_AsO_4_ (préparé au laboratoire, ASTM 01–775), Na_2_CO_3_ (Fluka, 71350) et AgNO_3_ (Prolabo, 21572) pris dans les rapports molaires Na:Ag:Mo:As égaux à 2:1:1:2 dans le but de synthétiser la série des composés à structure lamelaire A_2_MoO_2_As_2_O_7_ (A=Na et Ag; A=K (Zid *et al.*, 1996); A=Rb (Zid *et al.*, 1998)). Le mélange, finement broyé, est préchauffé à l'air à 673 K en vue d'éliminer NH_3_, H_2_O, CO_2_ et NO_2_. Il est ensuite porté jusqu'à une température de synthèse proche de la fusion, 808 K. Le mélange est alors abandonné à cette température pendant deux semaines pour favoriser la germination et la crissance des cristaux. Le résidu final a subi en premier un refroidissement lent (5°/jour) jusqu'à 758 K puis un second rapide (50°/h) jusqu'à la température ambiante. Des cristaux de couleur jaunatre, de taille suffisante pour les mesures des intensités, ont été séparés du flux par l'eau chaude. Une analyse qualitative au M.E.B.E. de type FEI Quanta 200 confirme la présence des différents éléments chimiques attendus: As, Mo, Ag, Na et l'oxygène.

### Refinement

L'analyse des Fouriers différences révèle l'existence de certains pics résiduels très proche des positions des ions Ag^+^. Dans l'affinement final et pour des raisons de neutralité électrique les taux d'occupation des cations Na^+^ et Ag^+^ ont été menés en utilisant la condition SUMP autorisée par le programme *SHELX* (Sheldrick, 2008). De plus les ellipsoïdes et les positions des ions Na^+^ ont été définis, moyennant respectivement les conditions EADP et EXYZ autorisée aussi par le programme *SHELX*, identiques à ceux des ions Ag^+^. En effet, les ellipsoïdes sont mieux définis. Les densités d'électrons max et min restants dans la Fourier-différence sont situées respectivements à 0,79 Å de As1 et à 1,81 Å de O10. Il en résulte la composition chimique finale, Ag_1,06 (1)_Na_1,94 (1)_Mo_3_AsO_13_ du nouveau matériau obtenu.

### Crystal data

**Table:**

AgNa_2_Mo_3_O_9_AsO_4_	*Z* = 2
*M**_r_* = 729.68	*F*(000) = 668
Triclinic, *P*1	*D*_x_ = 4.495 Mg m^−^^3^
Hall symbol: -P 1	Mo *K*α radiation, λ = 0.71073 Å
*a* = 8.1767 (8) Å	Cell parameters from 25 reflections
*b* = 9.7687 (9) Å	θ = 10–15°
*c* = 8.0451 (8) Å	µ = 8.50 mm^−^^1^
α = 99.49 (2)°	*T* = 298 K
β = 106.07 (2)°	Prism, yellow
γ = 113.29 (2)°	0.36 × 0.24 × 0.16 mm
*V* = 539.14 (19) Å^3^	

### Data collection

**Table:**

Enraf–Nonius CAD-4 diffractometer	2047 reflections with *I* > 2σ(*I*)
Radiation source: fine-focus sealed tube	*R*_int_ = 0.023
graphite	θ_max_ = 27.0°, θ_min_ = 2.4°
ω/2θ scans	*h* = −10→1
Absorption correction: ψ scan (North *et al.*, 1968)	*k* = −11→12
*T*_min_ = 0.098, *T*_max_ = 0.247	*l* = −10→10
2853 measured reflections	2 standard reflections every 120 min
2355 independent reflections	intensity decay: 1.1%

### Refinement

**Table:**

Refinement on *F*^2^	Primary atom site location: structure-invariant direct methods
Least-squares matrix: full	Secondary atom site location: difference Fourier map
*R*[*F*^2^ > 2σ(*F*^2^)] = 0.024	*w* = 1/[σ^2^(*F*_o_^2^) + (0.0204*P*)^2^ + 1.4359*P*] where *P* = (*F*_o_^2^ + 2*F*_c_^2^)/3
*wR*(*F*^2^) = 0.063	(Δ/σ)_max_ < 0.001
*S* = 1.09	Δρ_max_ = 0.95 e Å^−^^3^
2355 reflections	Δρ_min_ = −0.76 e Å^−^^3^
188 parameters	Extinction correction: *SHELXL97* (Sheldrick, 2008), Fc^*^=kFc[1+0.001xFc^2^λ^3^/sin(2θ)]^-1/4^
3 restraints	Extinction coefficient: 0.00141 (17)

### Special details

**Table:**

Geometry. All e.s.d.'s (except the e.s.d. in the dihedral angle between two l.s. planes) are estimated using the full covariance matrix. The cell e.s.d.'s are taken into account individually in the estimation of e.s.d.'s in distances, angles and torsion angles; correlations between e.s.d.'s in cell parameters are only used when they are defined by crystal symmetry. An approximate (isotropic) treatment of cell e.s.d.'s is used for estimating e.s.d.'s involving l.s. planes.
Refinement. Refinement of *F*^2^ against ALL reflections. The weighted *R*-factor *wR* and goodness of fit *S* are based on *F*^2^, conventional *R*-factors *R* are based on *F*, with *F* set to zero for negative *F*^2^. The threshold expression of *F*^2^ > σ(*F*^2^) is used only for calculating *R*-factors(gt) *etc*. and is not relevant to the choice of reflections for refinement. *R*-factors based on *F*^2^ are statistically about twice as large as those based on *F*, and *R*- factors based on ALL data will be even larger.

### Fractional atomic coordinates and isotropic or equivalent isotropic displacement parameters (Å^2^)

**Table:**

	*x*	*y*	*z*	*U*_iso_*/*U*_eq_	Occ. (<1)
Mo1	0.71234 (5)	0.14251 (5)	0.48605 (5)	0.00818 (11)	
Mo2	0.58834 (6)	0.75426 (5)	0.30104 (5)	0.00827 (11)	
Mo3	0.43503 (6)	0.22968 (5)	0.11436 (5)	0.00868 (11)	
As1	0.22233 (7)	0.82953 (5)	0.04824 (6)	0.00761 (12)	
Ag1	0.14302 (10)	0.43681 (8)	−0.09577 (9)	0.0213 (3)	0.524 (3)
Na1	0.14302 (10)	0.43681 (8)	−0.09577 (9)	0.0213 (3)	0.476 (4)
Ag2	0.84335 (15)	0.57626 (11)	0.60990 (13)	0.0320 (4)	0.391 (3)
Na2	0.84335 (15)	0.57626 (11)	0.60990 (13)	0.0320 (4)	0.609 (4)
Ag3	0.97290 (18)	−0.02400 (16)	0.74218 (18)	0.0194 (5)	0.147 (3)
Na3	0.97290 (18)	−0.02400 (16)	0.74218 (18)	0.0194 (5)	0.853 (4)
O1	0.2244 (5)	−0.0212 (4)	0.9676 (5)	0.0135 (7)	
O2	0.8795 (5)	0.3378 (4)	0.5567 (5)	0.0173 (8)	
O3	0.0460 (5)	0.6539 (4)	0.9111 (5)	0.0168 (8)	
O4	0.6979 (5)	0.7508 (4)	0.1170 (4)	0.0120 (7)	
O5	0.3912 (5)	0.5759 (4)	0.2330 (5)	0.0202 (8)	
O6	0.4357 (5)	0.8235 (4)	0.0849 (4)	0.0114 (7)	
O7	0.5640 (5)	0.1338 (4)	0.2611 (5)	0.0139 (7)	
O8	0.5097 (5)	0.8757 (4)	0.4424 (5)	0.0130 (7)	
O9	0.6297 (5)	0.4137 (4)	0.2142 (5)	0.0177 (8)	
O10	0.2813 (5)	0.2401 (5)	0.2173 (5)	0.0196 (8)	
O11	0.8223 (5)	0.0337 (4)	0.4165 (5)	0.0152 (7)	
O12	0.8183 (5)	0.1370 (4)	0.7539 (4)	0.0133 (7)	
O13	0.7650 (5)	0.7405 (4)	0.4636 (5)	0.0146 (7)	

### Atomic displacement parameters (Å^2^)

**Table:**

	*U*^11^	*U*^22^	*U*^33^	*U*^12^	*U*^13^	*U*^23^
Mo1	0.0074 (2)	0.0088 (2)	0.0082 (2)	0.00380 (16)	0.00317 (15)	0.00218 (15)
Mo2	0.0096 (2)	0.0090 (2)	0.00853 (19)	0.00560 (16)	0.00429 (15)	0.00358 (15)
Mo3	0.0106 (2)	0.0086 (2)	0.0085 (2)	0.00513 (16)	0.00467 (16)	0.00320 (15)
As1	0.0071 (2)	0.0071 (2)	0.0090 (2)	0.00346 (18)	0.00316 (18)	0.00267 (18)
Ag1	0.0186 (4)	0.0182 (4)	0.0256 (4)	0.0050 (3)	0.0110 (3)	0.0074 (3)
Na1	0.0186 (4)	0.0182 (4)	0.0256 (4)	0.0050 (3)	0.0110 (3)	0.0074 (3)
Ag2	0.0444 (7)	0.0255 (5)	0.0269 (5)	0.0217 (5)	0.0045 (4)	0.0116 (4)
Na2	0.0444 (7)	0.0255 (5)	0.0269 (5)	0.0217 (5)	0.0045 (4)	0.0116 (4)
Ag3	0.0142 (7)	0.0231 (8)	0.0215 (8)	0.0102 (6)	0.0056 (5)	0.0060 (6)
Na3	0.0142 (7)	0.0231 (8)	0.0215 (8)	0.0102 (6)	0.0056 (5)	0.0060 (6)
O1	0.0152 (17)	0.0105 (17)	0.0143 (17)	0.0060 (14)	0.0036 (14)	0.0065 (14)
O2	0.0177 (18)	0.0111 (17)	0.0166 (18)	0.0035 (15)	0.0031 (15)	0.0026 (14)
O3	0.0119 (17)	0.0117 (17)	0.0181 (18)	0.0012 (14)	0.0014 (15)	0.0015 (14)
O4	0.0155 (17)	0.0157 (17)	0.0116 (16)	0.0120 (15)	0.0062 (14)	0.0066 (14)
O5	0.0182 (19)	0.0136 (19)	0.027 (2)	0.0057 (16)	0.0079 (16)	0.0069 (16)
O6	0.0118 (16)	0.0171 (17)	0.0100 (16)	0.0093 (14)	0.0054 (14)	0.0066 (14)
O7	0.0164 (18)	0.0140 (17)	0.0117 (16)	0.0088 (15)	0.0035 (14)	0.0032 (14)
O8	0.0119 (17)	0.0153 (17)	0.0123 (16)	0.0067 (14)	0.0054 (14)	0.0037 (14)
O9	0.0188 (18)	0.0132 (18)	0.0201 (19)	0.0065 (15)	0.0087 (16)	0.0028 (15)
O10	0.024 (2)	0.025 (2)	0.0181 (19)	0.0152 (17)	0.0126 (16)	0.0087 (16)
O11	0.0165 (18)	0.0140 (18)	0.0186 (18)	0.0079 (15)	0.0102 (15)	0.0053 (15)
O12	0.0140 (17)	0.0224 (19)	0.0102 (16)	0.0129 (15)	0.0060 (14)	0.0071 (14)
O13	0.0159 (18)	0.0184 (19)	0.0119 (17)	0.0113 (16)	0.0035 (14)	0.0054 (14)

### Geometric parameters (Å, °)

**Table:**

Mo1—O2	1.727 (4)	Mo3—O6^iv^	2.249 (3)
Mo1—O11	1.758 (3)	As1—O3^v^	1.667 (4)
Mo1—O7	1.845 (3)	As1—O1^vi^	1.687 (3)
Mo1—O8^i^	2.004 (3)	As1—O6	1.713 (3)
Mo1—O12	2.107 (3)	As1—O12^i^	1.720 (3)
Mo1—O8^ii^	2.371 (4)	Ag1—O9^iv^	2.405 (4)
Mo2—O5	1.706 (4)	Ag1—O3^vii^	2.450 (4)
Mo2—O13	1.727 (3)	Ag1—O3^v^	2.539 (4)
Mo2—O8	1.917 (3)	Ag1—O5	2.574 (4)
Mo2—O4	1.933 (3)	Ag2—O3^viii^	2.318 (4)
Mo2—O6	2.212 (3)	Ag2—O13	2.321 (3)
Mo2—O11^iii^	2.455 (4)	Ag2—O2	2.446 (4)
Mo3—O10	1.710 (4)	Ag3—O1^viii^	2.322 (4)
Mo3—O9	1.722 (4)	Ag3—O12	2.381 (4)
Mo3—O7	1.959 (3)	Ag3—O11^ix^	2.390 (4)
Mo3—O4^iv^	1.961 (3)	Ag3—O10^x^	2.453 (4)
Mo3—O1^v^	2.225 (4)	Ag3—O13^ii^	2.511 (4)
			
O2—Mo1—O11	106.77 (17)	O13—Mo2—O11^iii^	82.93 (15)
O2—Mo1—O7	97.84 (16)	O8—Mo2—O11^iii^	68.41 (13)
O11—Mo1—O7	99.39 (16)	O4—Mo2—O11^iii^	83.59 (13)
O2—Mo1—O8^i^	108.96 (16)	O6—Mo2—O11^iii^	83.66 (13)
O11—Mo1—O8^i^	142.78 (15)	O10—Mo3—O9	103.12 (18)
O7—Mo1—O8^i^	85.84 (15)	O10—Mo3—O7	101.59 (16)
O2—Mo1—O12	89.08 (16)	O9—Mo3—O7	93.33 (16)
O11—Mo1—O12	90.26 (15)	O10—Mo3—O4^iv^	97.04 (16)
O7—Mo1—O12	165.90 (14)	O9—Mo3—O4^iv^	100.96 (16)
O8^i^—Mo1—O12	80.29 (13)	O7—Mo3—O4^iv^	153.26 (14)
O2—Mo1—O8^ii^	169.84 (14)	O10—Mo3—O1^v^	88.09 (16)
O11—Mo1—O8^ii^	72.75 (14)	O9—Mo3—O1^v^	168.35 (15)
O7—Mo1—O8^ii^	92.22 (14)	O7—Mo3—O1^v^	81.16 (13)
O8^i^—Mo1—O8^ii^	70.24 (15)	O4^iv^—Mo3—O1^v^	80.47 (14)
O12—Mo1—O8^ii^	80.79 (13)	O10—Mo3—O6^iv^	164.85 (16)
O5—Mo2—O13	104.45 (18)	O9—Mo3—O6^iv^	89.22 (15)
O5—Mo2—O8	98.74 (17)	O7—Mo3—O6^iv^	86.19 (13)
O13—Mo2—O8	103.37 (15)	O4^iv^—Mo3—O6^iv^	71.67 (12)
O5—Mo2—O4	106.41 (17)	O1^v^—Mo3—O6^iv^	80.22 (13)
O13—Mo2—O4	95.64 (15)	O3^v^—As1—O1^vi^	113.92 (17)
O8—Mo2—O4	143.46 (14)	O3^v^—As1—O6	107.68 (17)
O5—Mo2—O6	90.71 (16)	O1^vi^—As1—O6	110.61 (17)
O13—Mo2—O6	163.30 (15)	O3^v^—As1—O12^i^	106.89 (18)
O8—Mo2—O6	80.74 (13)	O1^vi^—As1—O12^i^	105.76 (17)
O4—Mo2—O6	72.99 (12)	O6—As1—O12^i^	111.99 (15)
O5—Mo2—O11^iii^	166.61 (15)		

Symmetry codes: (i) −*x*+1, −*y*+1, −*z*+1; (ii) *x*, *y*−1, *z*; (iii) *x*, *y*+1, *z*; (iv) −*x*+1, −*y*+1, −*z*; (v) *x*, *y*, *z*−1; (vi) *x*, *y*+1, *z*−1; (vii) −*x*, −*y*+1, −*z*+1; (viii) *x*+1, *y*, *z*; (ix) −*x*+2, −*y*, −*z*+1; (x) −*x*+1, −*y*, −*z*+1.
